# Plasma Neurofilament Light as a Biomarker of Neurological Involvement in Wilson's Disease

**DOI:** 10.1002/mds.28333

**Published:** 2020-10-20

**Authors:** Samuel Shribman, Carolin Heller, Maggie Burrows, Amanda Heslegrave, Imogen Swift, Martha S. Foiani, Godfrey T. Gillett, Emmanuel A. Tsochatzis, James B. Rowe, Alex Gerhard, Chris R. Butler, Mario Masellis, Fion Bremner, Alison Martin, Lynne Jung, Paul Cook, Henrik Zetterberg, Oliver Bandmann, Jonathan D. Rohrer, Thomas T. Warner

**Affiliations:** ^1^ Department of Clinical and Movement Neurosciences Reta Lila Weston Institute, UCL Queen Square Institute of Neurology London United Kingdom; ^2^ Dementia Research Centre, Department of Neurodegenerative Disease UCL Queen Square Institute of Neurology London United Kingdom; ^3^ Department of Neurodegenerative Disease UK Dementia Research Institute, UCL Queen Square Institute of Neurology London United Kingdom; ^4^ Department of Clinical Chemistry Northern General Hospital Sheffield United Kingdom; ^5^ UCL Institute for Liver and Digestive Health Royal Free Hospital and UCL London United Kingdom; ^6^ Department of Clinical Neurosciences University of Cambridge and Cambridge University Hospitals Trust Cambridge United Kingdom; ^7^ Division of Neuroscience and Experimental Psychology, Wolfson Molecular Imaging Centre University of Manchester Manchester United Kingdom; ^8^ Departments of Geriatric Medicine and Nuclear Medicine University of Duisburg‐Essen Duisburg Germany; ^9^ Department of Brain Sciences Imperial College London London United Kingdom; ^10^ Nuffield Department of Clinical Neurosciences University of Oxford Oxford United Kingdom; ^11^ Departamento de Neurología Pontificia Universidad Católica de Chile Santiago Chile; ^12^ Sunnybrook Health Sciences Centre Sunnybrook Research Institute, University of Toronto Toronto Ontario Canada; ^13^ Neuro‐Ophthalmology National Hospital for Neurology and Neurosurgery London United Kingdom; ^14^ Department of Clinical Biochemistry Southampton General Hospital Southampton United Kingdom; ^15^ Clinical Neurochemistry Laboratory Sahlgrenska University Hospital Mölndal Sweden; ^16^ Department of Psychiatry and Neurochemistry Institute of Neuroscience and Physiology, the Sahlgrenska Academy at the University of Gothenburg Mölndal Sweden; ^17^ Sheffield Institute for Translational Neuroscience, University of Sheffield Sheffield United Kingdom

**Keywords:** Wilson's disease, biomarkers, neurofilament light

## Abstract

**Background:**

Outcomes are unpredictable for neurological presentations of Wilson's disease (WD). Dosing regimens for chelation therapy vary and monitoring depends on copper indices, which do not reflect end‐organ damage.

**Objective:**

To identify a biomarker for neurological involvement in WD.

**Methods:**

Neuronal and glial‐specific proteins were measured in plasma samples from 40 patients and 38 age‐matched controls. Patients were divided into neurological or hepatic presentations and those with recent neurological presentations or deterioration associated with non‐adherence were subcategorized as having active neurological disease. Unified WD Rating Scale scores and copper indices were recorded.

**Results:**

Unlike copper indices, neurofilament light (NfL) concentrations were higher in neurological than hepatic presentations. They were also higher in those with active neurological disease when controlling for severity and correlated with neurological examination subscores in stable patients.

**Conclusion:**

NfL is a biomarker of neurological involvement with potential use in guiding chelation therapy and clinical trials for novel treatments. © 2020 University College London. *Movement Disorders* published by Wiley Periodicals LLC on behalf of International Parkinson and Movement Disorder Society

## 1 Introduction

Wilson's disease (WD) is an autosomal‐recessive disorder of copper metabolism with an estimated disease prevalence of 1 in 30,000.^1^ A movement disorder characterized by tremor, dystonia, parkinsonism, or a combination of these develops in those with neurological involvement, and psychiatric manifestations are also common.^2^ Presentations with neurological or psychiatric features are therefore classified as “neurological” whereas those with liver disease alone are classified as “hepatic”.^3^

Chelating agents prevent disease progression in the majority, and reduce neurological symptoms in some, but dosing regimens vary and these treatments can cause debilitating adverse effects and paradoxical worsening.^4^, ^5^ Monitoring involves measuring 24‐hr urinary copper output and calculating serum non‐ceruloplasmin‐bound copper.^5^ While consensus guidelines provide target ranges, these indices are method‐dependent and vary between individuals.^6^, ^7^ Moreover, there is no association between 24‐hr urinary copper output or serum non‐ceruloplasmin copper and neurological severity at presentation.^2^ Biomarkers of end‐organ damage are needed to improve neurological outcomes with chelation therapy and prepare for clinical trials of novel treatment strategies, such as gene therapy.^8^

Several neuronal or glial‐specific proteins are emerging as candidate biomarkers for other neurological diseases.^9^ Neurofilament light (NfL), also referred to as neurofilament light chain, is a cytoskeletal filament released during neuroaxonal injury that can be measured at low concentrations in plasma using single molecule array technology. Potential utility as a biomarker has been demonstrated in various neurodegenerative diseases.^10^, ^11^ In multiple sclerosis, serum concentrations are independently associated with clinical severity and disease status and reduce with escalation of disease‐modifying therapy.^9^ Tau, a microtubule‐stabilising protein, glial fibrillary acidic protein (GFAP), an intermediate filament, and ubiquitin carboxyl‐terminal hydrolase L1 (UCH‐L1), a cytoplasmic enzyme, can also be measured using similar methods.

These candidate biomarkers have not been studied in WD, with one exception: Lekomtseva and colleagues demonstrated that serum tau measured using an enzyme‐linked immunosorbent assay (ELISA) was higher in neurological presentations than controls but the relationship with neurological involvement was untested.^12^ We hypothesized that neuronal and glial‐specific proteins differentiate neurological from hepatic presentations, and indicate the severity of neurological involvement.

## 2 Patients and Methods

### 2.1 Study Design

We recruited 40 participants to a prospective study on WD. Consecutive patients attending neurology, hepatology, and metabolic outpatient clinics at the National Hospital for Neurology and Neurosurgery (NHNN) and Royal Free Hospital were invited to participate. Members of the WD Support Group UK research register were also sent an invitation letter via post. We included patients aged 16 years or over with diagnoses based on the Leipzig criteria.^3^ Exclusion criteria were any unrelated medical or psychiatric illness that would interfere with completing assessments and pregnancy.

Participants attended a baseline visit at NHNN for clinical assessments and venepuncture between January and December 2019. We used interviews during visits and subsequent review of records, where necessary, to confirm each participant's presentation at the time of their diagnosis using the international consensus on the phenotypic classification of WD^3^: those who had initially presented with neurological or psychiatric symptoms were classified as neurological presentations (n = 23). Those without were classified as hepatic presentations (n = 17), including asymptomatic individuals identified through family screening with previously abnormal liver function tests or hepatic imaging (n = 6).

We also subcategorized patients based on their recent neurological status. Those with a neurological presentation (n = 2), or a documented deterioration in neurological function related to non‐adherence (n = 3), in the preceding 6 months were considered to have active, as opposed to stable, neurological disease. We did not subcategorize patients according to their hepatic status.

Plasma samples from 38 age‐matched healthy controls were provided from the Genetic Frontotemporal Dementia Initiative cohort.^13^ These were family members of patients with MAPT, GRN, and C9orf72 mutations who had all undergone genetic testing and were non‐carriers.

The local ethics committees at each site approved the study and all participants provided written informed consent.

### 2.2 Procedures

A neurologist performed the Unified Wilson's Disease Rating Scale (UWDRS), a validated tool that includes components of the Unified Parkinson's Disease Rating Scale and Burke–Fahn–Marsden Dystonia Rating Scale, among others.^14^ Subscores for the neurological examination (UWDRS‐N, 18‐item), psychiatric assessment (UWDRS‐P, 19‐item), and functional impairment (UWDRS‐F, 9‐item) were recorded. A neuro‐ophthalmologist performed slit lamp examination to assess for Kayser–Fleischer (KF) rings.

Plasma samples from EDTA tubes were processed and stored at −80°C using the same standardized procedure for both patients and controls. NfL, tau, UCH‐L1, and GFAP concentrations were measured using Neurology 4‐Plex A kits on a Simoa HD‐X analyser (Quanterix, Billerica, MA, USA).

Serum ceruloplasmin and copper were determined using the immuno‐turbidimetric test (Beckman Coulter, Brea, CA, USA) and ICP‐MS (NexION 300, PerkinElmer, Waltham, MA, USA), respectively, and non‐ceruloplasmin copper (NCC) was calculated.^5^ Exchangeable copper (CuEXC), a labile fraction of serum copper bound to albumin and other peptides, was measured.^15^ Blood samples were centrifuged and stored at 4°C overnight. Equal volumes of EDTA 3 g/L were added to serum for 1 hr before ultrafiltration. Copper was measured in filtrates as above. Urinary copper output (UCu) was measured in 24‐hr collections while continuing medication. Aliquots were stored at −20°C before ICP‐MS (7700x system, Agilent, Santa Clara, CA, USA). Results from participants on zinc therapy or with liver transplants, in whom UCu is inherently lower, were excluded.

### 2.3 Statistical Analysis

We compared biomarkers and copper indices between the neurological, hepatic, and control groups using linear regression with age as a covariate, given NfL increases with age.^9^ We used receiver operating characteristic (ROC) curves to assess diagnostic performance in differentiating groups and calculated sensitivities and cut‐off values at 70%, 80%, and 90% specificities where the area under the curve (AUC) was increased (*P* < 0.05). We compared results between active and stable patients using linear regression with age and UWDRS‐N as covariates. We assessed associations between biomarkers, copper indices, and UWDRS subscores in stable patients using linear regression with age as a covariate and corrected for multiple testing using false discovery rate correction. We compared age and disease duration between patients and controls using unpaired *t* tests and assessed their association with biomarkers using Spearman's rank correlation coefficient. We used R3.6.0 for statistical analysis and GraphPad Prism 7 for graphs.

## 3 Results

Group demographic and clinical characteristics are summarised in Table 1. The most frequent examination findings in patients with neurological presentations were impaired finger taps (item 15, 87%), leg agility (item 20, 83%), arm and hand dystonia (item 23, 83%), oromandibular dystonia (item 11A, 78%), rapid alternating movements (item 16, 74%), handwriting (item 17, 74%), and speech (item 10, 70%).

**TABLE 1:** Group demographic and clinical characteristics, copper indices and biomarker results

Characteristic	Control (n = 38)	Hepatic (n = 17)	Neurologic (n = 23)	*P* ^a^	*P* ^b^	*P* ^c^
Age (yr) (mean (SD))	44 (13)	42 (15)	44 (14)	0.83	0.77	0.47
Sex (female) (n (%))	24 (63)	8 (47)	12 (52)	0.38	0.75	0.15
Disease duration (yr) (mean (SD))		20 (15)^d^	25 (16)^d^		0.37	0.56
Active disease status (n (%))		0 (0)	5 (22)			
KF rings present (n (%))		3 (23)^e^	14 (64)^e^			
Evidence of cirrhosis (n (%))		7 (41)^f^	10 (43)^f^			
Treatments (n (%))						
Penicillamine		9 (53)	17 (74)			
Trientine		5 (29)	4 (17)			
Zinc		1 (6)	0 (0)			
Combination		0 (0)	1 (4)			
Transplant		2 (12)	1 (4)			
UWDRS (median (IQR))						
UWDRS‐N		3 (0–4)	22 (14–37)			
UWDRS‐P		8 (3–13)	8 (4–15)			
UWDRS‐F		0 (0–1)	3 (2–9)			
Copper indices (median (IQR))						
NCC (μmol/L)		1.7 (1.4–2.3)	1.7 (1.4–2.6)		0.86	0.54
CuEXC (μmol/L)		0.6 (0.4–0.6)	0.5 (0.3–0.8)		0.71	0.13
UCu (μmol/24 hr)		4.2 (1.7–7.7)	5.0 (3.6–7.3)		0.28	<0.001
Biomarkers (median (IQR))						
NfL (ng/L)	7.6 (5.4–9.9)	7.0 (4.9–8.8)	8.7 (6.6–16.0)	0.005	0.005	0.048
Tau (ng/L)	1.4 (1.1–2.3)	1.4 (1.3–1.7)	1.8 (1.2–2.1)	0.57	0.13	0.57
GFAP (ng/L)	84 (65–136)	80 (67–90)	84 (65–136)	0.61	0.50	0.42
UCH‐L1 (ng/L)	23 (14–41)	23 (17–31)	23 (14–41)	0.014	0.06	0.87

Abbreviations: SD, standard deviation; KF, Kayser–Fleischer; IQR, interquartile range; UWDRS, Unified Wilson's Disease Rating Scale; ‐N, neurological examination subscore; ‐P, psychiatric subscore; ‐F, function subscore; NCC, non‐ceruloplasmin‐bound copper; CuEXC, exchangeable copper; UCu, urine copper; NfL, neurofilament light; GFAP, glial fibrillary acidic protein; UCH‐L1, ubiquitin carboxyl terminal hydrolase‐L1.

^a^ *P* values when comparing neurological and control groups.

^b^ *P* values when comparing neurological and hepatic groups.

^c^ *P* values when comparing active and stable patients.

^d^ Disease duration refers to symptom onset; six asymptomatic patients identified by family screening are excluded.

^e^ Four patients with hepatic and one patient with neurological presentations who were unable to have slit lamp examination and did not have Kayser–Fleischer rings at the bedside are excluded.

^f^ Evidence of cirrhosis determined by previous imaging and histopathology results. One participant had features of decompensated liver disease (ascites) during their research visit.

**FIG. 1:**
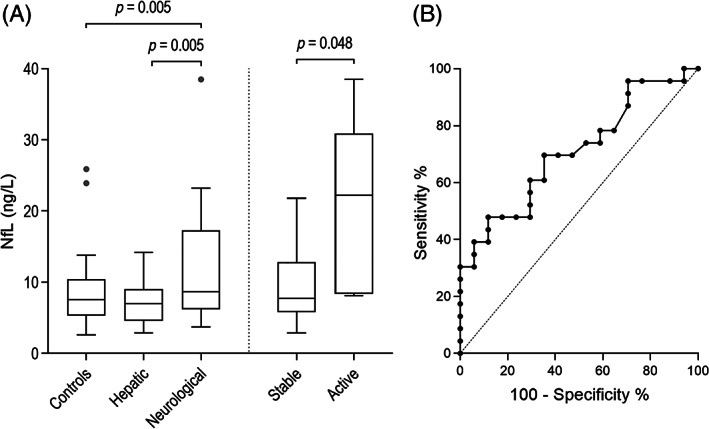
Group differences and the receiver operating characteristic (ROC) curve for neurofilament light (NfL). Box and whisker (Tukey) plots compare NfL between control, hepatic, and neurological groups and between active and stable patients (**A**). The ROC curve for NfL in differentiating neurological and hepatic groups (**B**).

There were no differences in copper indices between neurological and hepatic groups whereas NfL was higher in the neurological than hepatic and control groups. Copper indices and biomarker results are described in Table 1 and group differences and the ROC curve for NfL are depicted in Figure 1. The AUC for NfL in differentiating neurological and hepatic patients was 0.707 (*P* = 0.028). For 70%, 80%, and 90% specificities, the sensitivities were 61% (41–78%), 48% (29–67%), and 39% (22–59%) with cut‐off values of 8.4, 9.7, and 12.9 ng/L, respectively. UCH‐L1 was higher in neurological compared to control but not hepatic groups. There were no other group differences for other biomarkers.

NfL was higher in active compared to stable patients when controlling for neurological severity (22.2 vs. 7.7 ng/L, *P* = 0.048), as was UCu (16.1 vs. 4.0 μmol, *P* < 0.001). The highest NfL concentration (38.5 ng/L) and UWDRS‐N subscore (106) were in a newly‐diagnosed neurological patient with ongoing paradoxical worsening after starting penicillamine.

Associations between biomarkers, copper indices, and UWDRS subscores in stable patients are shown in Table S1. NfL was associated with UWDRS‐N subscores (β = 0.1, *P* = 0.003), tau was associated with UWDRS‐N (β = 0.02, *P* < 0.001) and UWDRS‐F (β = 0.06, *P* < 0.001) subscores, and UCH‐L1 was associated with UWDRS‐N (β = 0.95, *P* < 0.001) and UWDRS‐F (β = 2.70, *P* < 0.001) subscores. These associations persisted after correction for multiple testing. NfL concentrations were also correlated with NCC (β = 0.93, *P* = 0.04) but this association did not persist after correction for multiple testing.

NfL increased with age in controls (ρ = 0.74, *P* < 0.001) as expected, and there was no association between NfL and disease duration (ρ = 0.29, *P* = 0.10).

## 4 Discussion

We report differences in plasma NfL concentrations between neurological and hepatic presentations in a cohort of predominantly chronically‐treated WD patients. NfL and UCu were higher in the small number of cases with active neurological disease (after controlling for severity); however, UCu, like other copper indices, did not differ according to presentation.

The association of NfL with UWDRS‐N in stable patients adds further support for NfL as a potential biomarker of neurological involvement. It suggests that some participants had ongoing neuroaxonal injury despite treatment. We hypothesise that our definition of active disease might be too strict or some stable patients may have been non‐adherent or undertreated; the weak association between NfL and NCC supports this. Another possibility is that ongoing neuroaxonal injury in chronically‐treated patients does not solely depend on circulating copper levels; ATP7B is widely expressed in the brain,^16^ therefore dysfunctional intracellular copper handling within neurons might also influence neuronal vulnerability.

Importantly, NfL increases with age and age‐specific reference ranges are not yet available.^9^ Group differences were clearer when evaluated with linear regression than with ROC curve analysis because age was a covariate, and our cohort included older adults. NfL may detect neurological involvement at initial presentation with greater sensitivity and specificity than our results suggest given WD usually presents in adolescence or early adulthood. Evidence from a cohort of presymptomatic patients with Huntington's disease indicates that NfL is a highly sensitive marker of neurodegeneration in young people; concentrations in plasma increased approximately 24 years before predicted disease onset.^17^

The association of tau and UCH‐L1 with UWDRS subscores in the absence of differences between active and stable patients suggests these reflect more chronic neurological involvement. The lack of association between GFAP, a glial‐specific protein, and UWDRS subscores suggests that reactive gliosis is not prominent in chronically‐treated patients.

The small size of our cohort, specifically the inclusion of only five patients with active disease, is the main limitation of this study: any lack of differences should be interpreted with care. The next step is to study our cohort prospectively. NfL will also need to be investigated in larger, independent cohorts of recently‐diagnosed patients or as an exploratory outcome measure in clinical trials for WD.

Our findings may have important implications for WD monitoring given neurological disease activity is currently determined by examination alone.^5^ Unlike most neurodegenerative diseases, treatments affecting disease course are already established in WD, and the introduction of novel monitoring approaches could improve neurological outcomes in the short term. We predict a role for NfL in monitoring both “de‐coppering” and maintenance phases of WD treatment. NfL might help identify which treatments and doses optimize neurological recovery and indicate paradoxical worsening.

## Authors' Roles

(1) Research Project: A. Conception, B. Organization, C. Execution; (2) Statistical Analysis: A. Design, B. Execution, C. Review and Critique; (3) Manuscript Preparation: A. Writing of the First Draft, B. Review and Critique.

S.S.: 1A, 1B, 1C, 2A, 2B, 3A

C.H.: 1B, 1C, 3B

M.B.: 1C

A.H.: 1B, 1C

I.S.: 1C

M.S.F.: 1C

G.T.G.: 1A, 1B, 1C, 3B

E.A.T.: 1A, 1C, 3B

J.B.R.: 1C, 3B

A.G.: 1C

C.R.B.: 1C

M.M.: 1C, 3B

F.B.: 1C

A.M.: 1C

L.J.: 1B, 1C

P.C.: 1A, 1B, 3B

H.Z.: 1A, 1B, 2A, 3B

O.B.: 1A, 2A, 2C, 3B

J.D.R.: 1A, 1B, 1C, 2A, 2C, 3B

T.T.W.: 1A, 1B, 1C, 2A, 2C, 3B

## Financial Disclosures

H.Z. has served on scientific advisory boards for Denali, Roche Diagnostics, Wave, Samumed, and CogRx; has given lectures in symposia sponsored by Fujirebio, Alzecure, and Biogen; and is a co‐founder of Brain Biomarker Solutions in Gothenburg AB, a GU Ventures‐based platform company at the University of Gothenburg. M.M. has served as a board member at Current Pharmacogenomics and Personalized Medicine and at scientific advisory boards for Arkuda Therapuetics, Ionis, and Alector. He has received grants from the Canadian Institutes of Health Research, Ministry of Economic Development and Innovation of Ontario, Ontario Brain Institute, Sunnybrook AFP Innovation Fund, Alzheimer's Drug Discovery Foundation, Brain Canada, Heart and Stroke Foundation Centre for Stroke Recovery, Weston Brain Institute, Roche, Washington University, Axovant, Novartis, and Alector. He has received personal fees from Henry Stewart Talks and Alector. JR has recieved grant support from The Wellcome Trust and Medical Research Council and NIHR Cambridge Biomaedical Research Centre, Lilly, AZ‐Medimmune and Janssen. He has served on advisory boards for Biogen, Astex, WAVE, SCHealth and Ascbeuron. The remaining authors have no financial disclosures.

## Supporting information

**Table S1** Associations between biomarkers, copper indices and Unified Wilson's Disease Rating Scale (UWDRS) subscores
