# Supplementary material for: Plasma Neurofilament Light as a Biomarker of Neurological Involvement in Wilson's Disease
Source: Mov Disord. 2020 Oct 20;36(2):503–8. doi: 10.1002/mds.28333 (PMC8436757; doi:10.1002/mds.28333)
Supplement: Supplementary file 1 — Table S1 Associations between biomarkers, copper indices and Unified Wilson's Disease Rating Scale (UWDRS) subscores [file MDS-36-503-s001.docx]

**Supplementary Table** **1** Associations between biomarkers, copper indices and UWDRS subscores

| **Characteristic** | | **NfL** | **Tau** | **GFAP** | **UCH-L1** | **NCC** | **CuEXC** | **UCu** |
| --- | --- | --- | --- | --- | --- | --- | --- | --- |
| UWDRS-N | |  |  |  |  |  |  |  |
| β | | 0.10 | 0.02 | -0.26 | 0.95 | 0.00 | 0.01 | -0.06 |
| P value | | 0.003^a^ | <0.001^a^ | 0.74 | <0.001^a^ | 0.69 | 0.55 | 0.11 |
| UWDRS-P | |  |  |  |  |  |  |  |
| β | | -0.03 | 0.02 | -2.46 | -0.33 | 0.00 | 0.00 | 0.00 |
| P value | | 0.79 | 0.21 | 0.22 | 0.64 | 0.63 | 0.97 | 0.98 |
| UWDRS-F | |  |  |  |  |  |  |  |
| β | | 0.21 | 0.06 | -0.31 | 2.70 | -0.01 | 0.04 | -0.12 |
| P value | | 0.06 | <0.001^a^ | 0.90 | <0.001^a^ | 0.46 | 0.35 | 0.31 |
| NCC | |  |  |  |  |  |  |  |
| β | | 0.93 | -0.04 | -1.47 | 5.76 |  |  |  |
| P value | | 0.036 | 0.67 | 0.99 | 0.12 |  |  |  |
| CuEXC | |  |  |  |  |  |  |  |
| β | | 1.81 | -0.04 | -44.89 | 18.78 |  |  |  |
| P value | | 0.47 | 0.67 | 0.46 | 0.35 |  |  |  |
| UCu |  |  |  |  |  |  |  |  |
| β | | -0.29 | -0.03 | -3.98 | -1.20 |  |  |  |
| P value | | 0.07 | 0.35 | 0.32 | 0.37 |  |  |  |

Abbreviations: β = coefficient; NfL = neurofilament light; GFAP = glial fibrillary acidic protein; UCH-L1 = ubiquitin carboxyl terminal hydrolase-L1; NCC = non-ceruloplasmin-bound copper; CuEXC = exchangeable copper; UCu = urine copper; UWDRS = Unified Wilson’s Disease Rating Scale; -N = neurological examination subscore; -P = psychiatric subscore; -F = function subscore.

^a^ Significant after correction for multiple testing (false discovery rate adjusted P value < 0.05).
